# Ideal Cardiovascular Health in the Oldest-Old and Centenarians and Its Association With Disability and Health-Related Quality of Life

**DOI:** 10.3389/fcvm.2021.603877

**Published:** 2021-08-20

**Authors:** Miao Liu, Fuyin Kou, Shanshan Yang, Shengshu Wang, Yao He, Wuping Zhang

**Affiliations:** ^1^Department of Statistics and Epidemiology, Graduate School of Chinese People's Liberation Army General Hospital, Beijing, China; ^2^Health Service Department, Chinese People's Liberation Army General Hospital, Beijing, China; ^3^The 1st Medical Center, Department of Disease Prevention and Control, Chinese People's Liberation Army General Hospital, Beijing, China; ^4^National Clinical Research Center for Geriatrics Diseases, Beijing Key Laboratory of Aging and Geriatrics, State Key Laboratory of Kidney Diseases, Institute of Geriatrics, Second Medical Center of Chinese People's Liberation Army General Hospital, Beijing, China; ^5^Foreign Language Department, Graduate School, Graduate School of Chinese People's Liberation Army General Hospital, Beijing, China

**Keywords:** ideal cardiovascular health, disability, quality of life, centenarian, epidemiology

## Abstract

**Objective:** This study aimed to investigate the distribution of ideal cardiovascular health (ICH) indicators among the oldest-old and centenarians and explore their relationships with disability and health-related quality of life of this population.

**Methods:** One thousand two centenarians from China Hainan Centenarian Cohort Study and 798 oldest-old from the China Hainan Oldest-old Cohort study were the target subjects in this analysis. ICH status, disability, and health-related quality of life of study subjects were assessed.

**Findings:** The median value of ICH indicators among centenarians and the oldest-old is 4 (4–5) and 3 (3–5), respectively. The ICH indicators with the highest percentage of ideal level/status are fasting plasm glucose (FPG) (90.2% of study subjects are at the ideal level), BMI (89.8% of study subjects are at the ideal level), and smoking (89.4% of study subjects are at the ideal status). The disability rates of basic activities of daily living (BADL) and instrumental activities of daily living (IADL) decrease with the increasing number of ICH indicators. The EQ VAS and EQ-5D score show an increasing trend along with the increasing number of ICH indicators (*p* < 0.05). After adjusting related covariates, the risk of disability and lower health-related quality of life decreased gradually as the number of ICH metrics increased (*p* < 0.05).

**Interpretation:** The ICH metrics of centenarians and oldest-old were at a relatively good level, and there was a strong and independent relationship between the number of ICH indicators and disability as well as the lower health-related quality of life.

## Introduction

Cardiovascular diseases are a major public health problem affecting not only health but also health-related quality of life all around the world (1, 2). It is the leading cause of mortality in China and the cardiovascular disease burden increases as the population ages. The latest burden of disease report in China shows that ischemic heart diseases and stroke are the top causes of death (3). Understanding and identifying health risk factors is particularly important for the prevention and control of cardiovascular diseases. In 2010, the American Heart Association (AHA) developed new criteria to define risk factors for cardiovascular disease and to assess whether the cardiovascular system is in an ideal state using the ideal cardiovascular health (ICH) metrics. ICH metrics consists of seven factors and plays a major role in assessing cardiovascular health risks (4). Numerous studies have shown that keeping people in ICH status is the cornerstone to reducing the cardiovascular disease incidence (5, 6). It has also been pointed out that the status of ICH is not only related with cardiovascular diseases, but also related with diabetes, depression, cancer, and other diseases or health outcomes (7–9). Better ICH status is closely related to a higher health-related quality of life and a lower disability rate (10–12).

However, most current studies are conducted upon adults or young elderly (11, 13). There is a lack of understanding of ICH status for the elderly who are above 80 years old. Most of the studies on the elderly population focus on hospitalized patients or the elderly from senior care facilities; little knowledge is known about the general elderly population (14). Therefore, this study aims to understand the characteristics of ICH metrics distribution and their relationship with disability and health-related quality of life in the centenarian and the oldest-old population by analyzing the information collected from China Hainan Centenarian Cohort Study (CHCCS) and China Hainan Oldest-old Cohort study (CHOCS).

## Study Design and Methods

### Study Population

The study subjects were recruited from the CHCCS and CHOCS. The framework of study design has been published in the International Journal of Epidemiology (15). From January 2014 through to December 2016, we surveyed a total number of 1,002 eligible centenarians who were more than 100 years old from 18 cities and counties in Hainan province, which is the province with the highest proportion of longevity population in China. Meanwhile, a multi-stage sampling method was used to select a group of oldest-old subjects who were 80–100 years old. Probability proportional to size (PPS) sampling was used to determine the locations from the district level then to the village and community level. A total number of 798 oldest-old who were between 80 and 100 years old were included.

### Investigation Method

We had a face-to-face interview with each study subject. All the interviewers were medical and nursing staff from Hainan Hospital affiliated to the Chinese PLA General Hospital. A health questionnaire was used to collect detailed demographic and sociological characteristics, disease history, family history, lifestyle, disability, and health-related quality of life for all subjects. Height, weight, and blood pressure were measured by trained nurses. The body length of subjects with kyphosis was measured instead of height. Fasting venous blood (over 8 h of fasting) was collected during 7–8 a.m. and sent to the department of biochemistry, Hainan Hospital, within 4 h, to test the level of total cholesterol (TC), triglyceride (TG), high density lipoprotein cholesterol (HDL-C), low density lipoprotein cholesterol (LDL-C), and fasting blood glucose (FPG).

### Definitions

The age of a subject is calculated as the differences between the date of interview and their date of birth (validated through the subject's ID card information) in years. Centenarians are subjects who were more than 100 years old on the day of survey. The oldest-old are the subjects who were 80–99 years old on the day of the survey. The ethnicity of a subject is classified as either Han or minority. Marital status is divided as widowed or not (including married, divorced, or other). The education level is classified according to the total years of education: illiteracy (0 years), primary school (0–6 years), and middle school and above (7–12years). Body mass index (BMI) is calculated as weight in kilograms divided by the square of height in square meters. Alcohol drinking status is classified into three categories: current, former, and never. Coronary heart disease and stroke history is determined from their medical records output from Grade II and III hospitals.

The assessment of disability includes two aspects: basic ability of daily life (BADL) and instrumental ability of daily life (IADL). The BADL is measured by the Barthel index, which includes 10 items (including feeding, bathing, dressing, bowel and bladder control, toilet use, chair transferring, dressing, stair climbing, and ambulating) with a total score of 100. The IADL is measured by Lawton scale, which includes eight items (namely phone use, cooking, washing, taking medication, shopping, housekeeping, transportation, and financial ability) with a total score of eight. The assessment outcome of disability used in this study is presented in two ways-two levels: complete self-care and disability; and four levels: complete self-care, mild disability, moderate disability, and severe disability. The level of BADL and IADL is shown in Appendix Table 1 (16–18).

Quality of life was assessed by the EQ-5D-3L scale (which was developed by EuroQol Group). The EQ-5D-3L is composed of two parts: a self-reported scale with five elements, namely mobility, self-care, pain/discomfort, daily activities, and anxiety/depression, and a visual analog scale (VAS) which reflects current overall health on a scale from 0 to 100. The EQ-5D score is calculated using the Chinese value sets (19).

ICH metrics are defined by the AHA expert panel (4) and modified to the characteristics of the Chinese population. The ICH metrics include seven items: smoking status, BMI, physical activity level, diet, blood pressure level, total cholesterol level, and FPG level. For each ICH metric, there are three levels of cardiovascular health status: poor, intermediate, and ideal (Appendix Table 2). The quality of a diet is evaluated by the consumption level of fish, vegetables, red meat, eggs, and preference for salty food. ICH is assessed among participants who have no history of coronary heart diseases or stroke.

### Ethics Approval and Consent to Participate

This study was approved by the Biomedical Ethics Committee of Chinese PLA general hospital. Written informed consent was obtained voluntarily from each participant.

### Statistical Analysis

Survey information was entered and stored using statistical software Epidata3.1. The mean value and standard deviation (SD) were calculated for each continuous variable and the percentage (*n* %) distribution was calculated for each categorical variable. Variance analysis or a non-parametric test was used to compare continuous variables. Chi-square test was used to compare categorical variables. Multivariable logistic regression was used to predict the OR (95% CI) of ICH associated with each dependent variable. The ICH status of five levels (0–2, 3, 4, 5, and 6–7) was used as the outcome variable. Covariates included gender, age, ethnicity, culture, marital status, and alcohol consumption. ADL disability, moderate to severe ADL disability, IADL disability, moderate to severe IADL disability, and low EQ-5D were used as binary dependent variables, respectively.

## Results

There are 1,002 centenarians and 798 oldest-old participants in this study. The average age is 102.77 and 84.99 years for each group. Percentages of female participants, widowed participants, and illiterate participants in centenarians are all higher than in the oldest-old. The average weight, waist circumference (WC), and average level of, DBP, TC, TG, and LDL-C are all lower in centenarians than in oldest-old, whereas systolic blood pressure and fasting blood sugar level are higher. Results are shown in Table 1.

**Table 1:** General characteristics of samples.

**Characteristics**	**Sample**		
	**CHCCS**	**CHONS**	***p***
**Mean ± SD**			
Age (yrs)	102.77 ± 2.75	84.99 ± 4.10	<0.001
Weight (kg)	37.85 ± 7.66	47.54 ± 10.45	<0.001
WC (cm)	75.27 ± 8.79	79.17 ± 9.80	<0.001
SBP (mmHg)	151.32 ± 22.67	147.92 ± 23.32	0.002
DBP (mmHg)	75.10 ± 11.90	80.03 ± 11.83	<0.001
TC (mmol/l)	4.67 ± 0.99	5.08 ± 1.07	<0.001
TG (mmol/l)	1.17 ± 0.65	1.27 ± 0.67	0.003
HDL-C (mmol/l)	1.44 ± 0.39	1.43 ± 0.40	0.360
LDL-C (mmol/l)	2.80 ± 0.79	3.09 ± 0.90	<0.001
FPG (mmol/l)	5.15 ± 1.44	4.82 ± 2.27	<0.001
***n*** **(%)**			
Gender			<0.001
Male	180 (18.0)	320 (40.1)	
Female	882 (82.0)	478 (59.9)	
Nationality			0.156
Han	883 (88.1)	720 (90.2)	
Minority	119 (11.9)	78 (9.8)	
Marriage status			<0.001
Widowed	836 (84.1)	343 (43.3)	
Married/divorced/others	158 (15.9)	410 (56.7)	
Education level			0.880
Illiteracy	915 (91.3)	605 (75.8)	
Primary school	67 (6.7)	119 (14.9)	
Middle school or above	20 (2.0)	74 (9.3)	
Alcohol drinking			0.152
Current	49 (4.9)	65 (8.1)	
Former	81 (8.1)	62 (7.8)	
Never	872 (87.0)	671 (84.1)	
Smoking			<0.001
Current	35 (3.5)	79 (9.9)	
Former	34 (3.4)	42 (5.3)	
Never	933 (93.1)	677 (84.8)	
Previous cardiovascular disease	41 (4.1)	63 (7.9)	0.001
Previous stroke	9 (0.9)	14 (1.8)	0.108

*CHCCS, China Hainan Centenarian Cohort Study; CHOCS, China Hainan Oldest-old Cohort Study; SD, standard deviation; SBP, systolic blood pressure; DBP, diastolic blood pressure; TC, total cholesterol; TG, triglyceride; LDL-C, low density lipoprotein cholesterol; HDL-C, high density lipoprotein cholesterol; FPG, fasting plasma glucose*.

### Distribution of ICH Metrics in Centenarians and Oldest-Old

**Figure 1:**
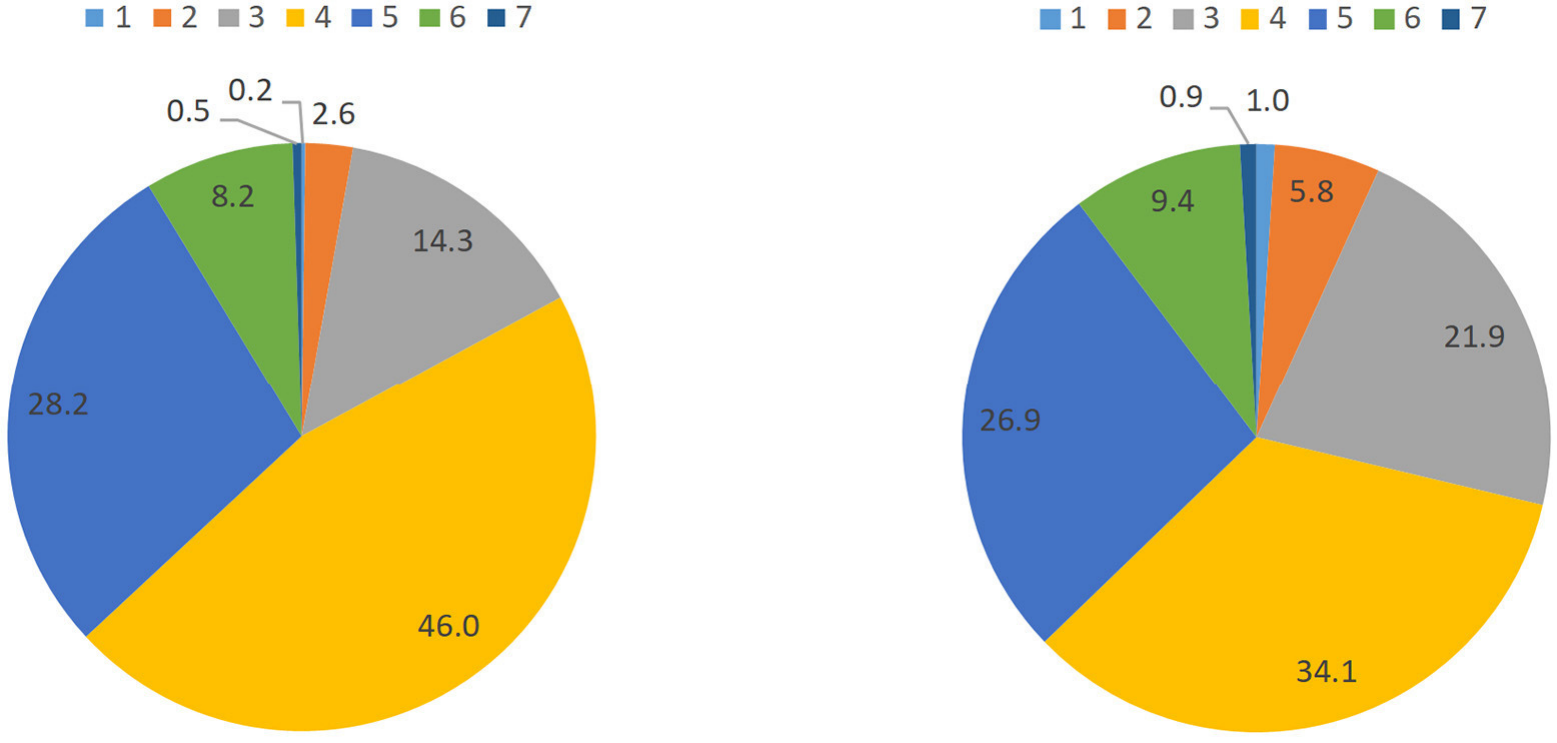
Distribution of ICH in centenarians and oldest-old.

The distribution of ICH metrics in centenarians and oldest-old is presented in Table 2 and Figure 1. The distribution of ICH metrics among centenarians is more centralized than that in the oldest-old. The median levels of ICH metrics among centenarians and oldest-old are 4 (4–5) and 3 (3–5), respectively. 8.7% of the centenarians have more than 6 ICH metrics at the ideal levels; 74.2% have 4–5 ICH metrics at the ideal levels. 10.3% of the oldest-old have more than 6 ICH metrics at the ideal levels, while 82.9% have 3–5 ICH metrics at the ideal levels. FPG (90.2%), BMI (89.8%), and smoking (89.4%) have the highest percentage of being at the ideal level while blood pressure (8.3%) and BMI (1.9%) had the lowest percentage (8.3%) (Table 2). Centenarians have a higher percentage of being at the ideal levels in ICH metrics of smoking (93.1 vs. 84.8%), BMI (96.3 vs. 89.8%), and TC (73.7 vs. 59.1%), meanwhile the oldest-old have a higher percentage of being at the ideal levels in physical activity (45.0 vs 32.8%) and healthy diet (41.2 vs. 32.4%).

**Table 2:** Distribution of ICH components.

	**CHCCS**				**CHONS**				p^*^	**Total**
	**Male**	**Female**	***P***	**Subtotal**	**Male**	**Female**	***p***	**Subtotal**		
Smoking			<0.001				<0.001		<0.001	
Ideal	75.6	97.0		93.1	67.2	96.7		84.8		89.4
Intermediate	10.6	1.8		3.4	11.3	1.3		5.3		4.2
Poor	13.9	1.2		3.5	21.6	2.1		9.9		6.3
BMI			0.068				0.111		<0.001	
Ideal	95.0	96.6		96.3	80.0	82.8		81.7		89.8
Intermediate	5.0	2.6		3.0	17.5	13.0		14.8		8.2
Poor	0.0	0.9		0.7	2.5	4.2		3.5		1.9
Physical activity			0.010				0.001		<0.001	
Ideal	42.2	30.8		32.8	52.2	40.2		45.0		38.2
Intermediate	11.7	11.8		11.8	15.0	13.4		14.0		12.8
Poor	46.1	57.4		55.4	32.8	46.4		41.0		49.0
Healthy diet			0.735				0.487		<0.001	
Ideal	34.4	32.0		32.4	44.1	41.2		42.4		36.8
Intermediate	61.1	62.5		62.3	53.1	54.6		54.0		58.6
Poor	4.4	5.5		5.3	2.8	4.2		3.6		4.6
Total cholesterol			0.144				0.001		<0.001	
Ideal	79.4	72.4		73.7	64.7	55.4		59.1		67.2
Intermediate	15.6	20.2		19.4	26.9	26.8		26.8		22.7
Poor	5.0	7.4		7.0	8.4	17.5		14.0		10.1
Blood pressure			0.013				0.001		0.006	
Ideal	11.1	6.6		7.4	13.8	6.5		9.4		8.3
Intermediate	24.4	18.7		19.8	26.3	23.6		24.7		21.9
Poor	64.6	74.7		72.9	60.0	69.9		65.9		69.8
FPG			0.985				0.589		0.998	
Ideal	87.8	87.6		90.5	90.6	89.1		89.7		90.2
Intermediate	2.8	2.9		3.5	1.9	1.5		3.6		0.7
Poor	9.4	9.5		6.0	7.5	9.4		6.6		9.1
Number of ICH			0.714				0.902		< .001	
0	0.0	0.0		0.0	0.0	0.0		0.0		0.0
1	0.6	0.1		0.2	1.3	0.8		1.0		0.6
2	2.2	2.7		2.6	6.6	5.2		5.8		4.0
3	17.8	13.5		14.3	22.2	21.8		21.9		17.7
4	39.4	47.4		46.0	30.3	36.6		34.1		40.7
5	28.9	28.1		28.2	29.1	25.5		26.9		27.7
6	10.0	7.8		8.2	9.4	9.4		9.4		8.7
7	1.1	0.4		0.5	1.3	0.6		0.9		0.7

* *p for comparisons between CHCCS and CHONS*.

*CHCCS, China Hainan Centenarian Cohort Study; CHOCS, China Hainan Oldest-old Cohort Study; ICH, ideal cardiovascular health*.

### The Relationship Between the Level of ICH Metrics and Disability

39.1% of total participants are complete BADL self-care and 12.8% are IADL self-care. Disability rate is much higher in centenarians than in the oldest-old. Male participants have better ADL than female, both in centenarians and oldest-old (*p* < 0.05) (Appendix Table 3).

**Figure 2:**
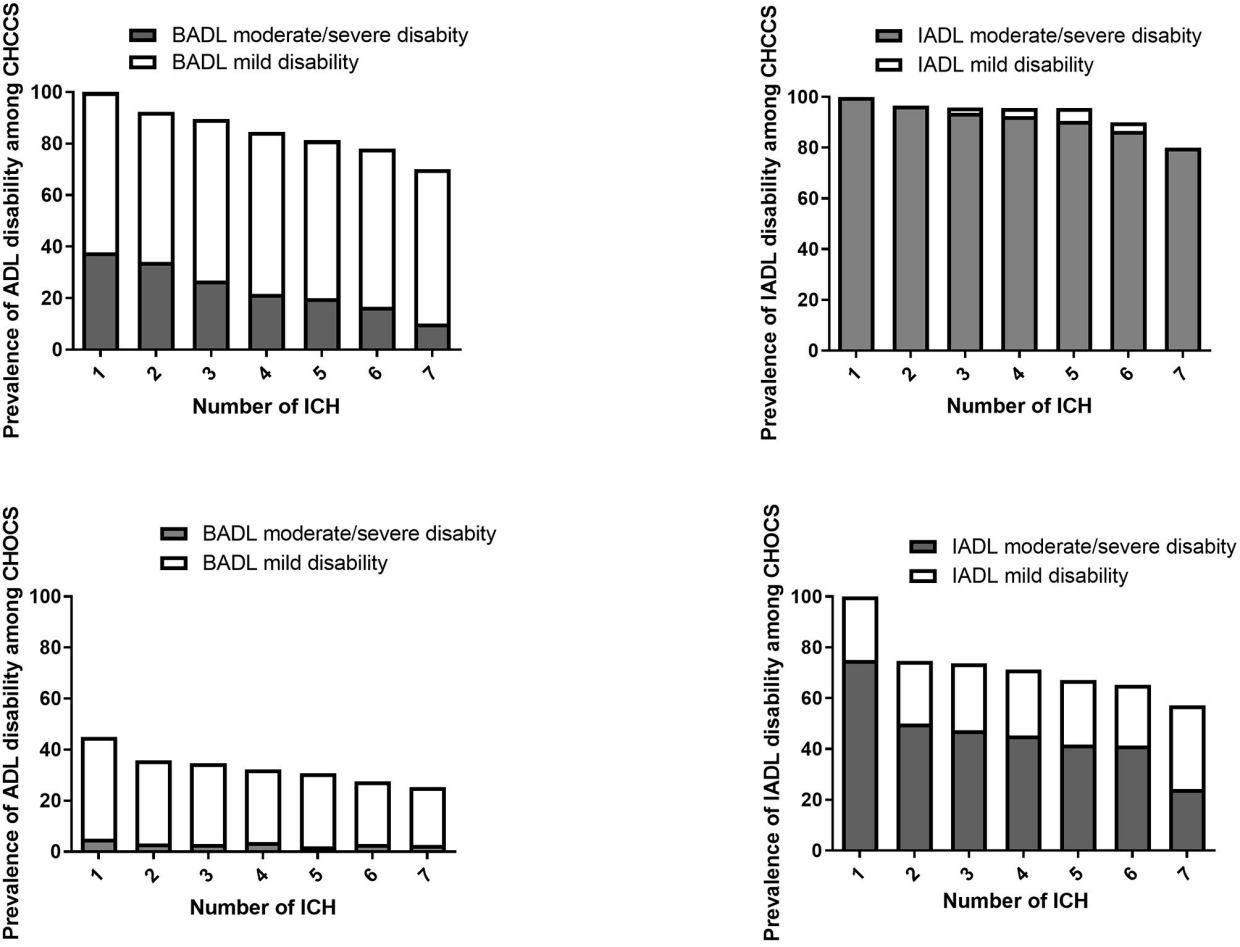
BADL and IADL disability with number of ICH metrics.

Disability rates of BADL and IADL decrease as the number of ICH metrics increases (Figure 2). Among the centenarians, BADL disability decreases from 100.0 to 73.2% as the number of ICH increases from 1 to 7. The IADL disability rate decreases from 100 to 80.0% and is largely driven by subjects with moderate to severe IADL disability. The disability rates among the oldest-old also show a downward trend as the number of ICH metrics increases.

### The Relationship Between the Number of ICH Metrics and Health-Related Quality of Life

The EQ VAS and EQ-5D score are higher in the oldest-old than in centenarians (*p* < 0.05). The EQ VAS and EQ-5D score show an increasing trend with the number of ICH metrics increases (*p* < 0.05) (Table 3).

**Table 3:** Distribution of ICH and association with EQ-5D.

	**Number of ICH**							***p***
	**1**	**2**	**3**	**4**	**5**	**6**	**7**	
**CHCCS**								
EQ VAS (mean ± SD)	58.0 ± 9.9	59.0 ± 17.7	59.0 ± 16.3	60.3 ± 15.1	63.6 ± 15.5	67.1 ± 14.7	70.0 ± 15.6	0.002
EQ-5D score (mean ± SD)	0.58 ± 0.05	0.58 ± 0.06	0.59 ± 0.07	0.60 ± 0.07	0.69 ± 0.05	0.75 ± 0.03	0.78 ± 0.03	<0.001
EQ-5D score <1 (%)	100	96.2	90.2	90	83.4	75.6	60	<0.001
BADL score	75.0 (50.0–90.0)	80.0 (55.0–95.0)	85 (47.5–95.0)	80.0 (62.5–95.0)	90.0 (90–100.0)	90.0 (55.0–95.0)	95.0 (85.0–100.0)	<0.001
IADL score	1.5 (0.0–2.0)	1.0 (0.0–3.0)	1.0 (0.0–2.0)	1.0 (0.0–3.0)	2.0 (0.0–4.0)	3.0 (1.0–5.0)	2.0 (0.5–3.5)	<0.001
BADL disability	100.0	92.3	89.5	84.6	81.3	80.0	73.2	0.001
BADL moderate/severe disability	50.0	37.8	34.1	26.9	21.6	20.0	8.5	<0.001
IADL disability	100.0	98.7	98.6	98.7	97.5	92.3	80.0	0.025
IADL moderate/severe disability	100.0	96.5	92.3	92.4	90.5	86.6	80.0	0.006
**CHOCS**								
EQ VAS (mean ± SD)	66.3 ± 8.8	68.2 ± 10.9	68.4 ± 10.5	68.5 ± 10.5	71.3 ± 9.9	73.5 ± 10.6	73.6 ± 8.0	0.001
EQ-5D score (mean ± SD)	0.83 ± 0.03	0.86 ± 0.03	0.87 ± 0.03	0.89 ± 0.02	0.90 ± 0.03	0.90 ± 0.03	0.92 ± 0.03	0.017
EQ-5D score <1(%)	62.5	54	48.2	44	42.5	36	34.1	0.022
BADL score [median (IQR)]	92.5 (8.0–95.0)	100.0 (95.0–100.0)	100.0 (95.0–100.0)	100.0 (95.0–100.0)	100.0 (95.0–100.0)	100.0 (950.0–100.0)	100.0 (95.0–100.0)	0.019
IADL score [median (IQR)]	4.0 (1.5–5.8)	5.5 (3.8–8.0)	6.0 (4.0–8.0)	6.0 (3.0–7.0)	6.0 (4.0–8.0)	6.0 (5.0–8.0)	7.0 (4.0–8.0)	0.047
BADL disability (%)	87.5	34.8	35.3	32.6	30.2	28.6	22.7	0.001
BADL moderate/severe disability (%)	12.5	5.6	4.0	3.7	2.0	0.0	0.0	0.035
IADL disability (%)	100.0	77.2	73.0	72.0	69.6	65.3	57.1	0.027
IADL moderate/severe disability (%)	75.0	50.0	47.4	45.2	41.7	41.3	14.3	0.026

*CHCCS, China Hainan Centenarian Cohort Study; CHOCS, China Hainan Oldest-old Cohort Study; ICH, ideal cardiovascular health; IQR, inter quartile range; BADL, basic ability of daily life; IADL, instrumental ability of daily life*.

### Multivariable Analysis

Multivariable regression was used to analyze the ORs of ICH metrics for disability and lower health-related quality of life (defined as having the EQ-5D score < 1). Subjects who had been diagnosed with coronary diseases or stroke were removed from the association analysis. After adjusting related covariates, the risk of disability and lower health-related quality of life decreased gradually with the increase of ICH numbers (*p* < 0.05). When using the moderate to severe disability as the dependent variables, the ORs also decrease as the number of ICH metrics increases (*p* < 0.05) (Table 4).

**Table 4:** Multivariable analysis of ICH with disability and health-related quality of life.

**Variables**	**Number of ICH, OR (95%CI)**					
	**≤2**	**3**	**4**	**5**	**≥6**	***p***
**CHCCS**						
BADL disability	1.00 (Ref)	0.82 (0.48–2.72)	0.66 (0.19–2.24)	0.52 (0.15–1.79)	0.44 (0.29–1.41)	0.020
BADL moderate/severe disability	1.00 (Ref)	0.80 (0.46–2.73)	0.56 (0.23–2.45)	0.44 (0.24–3.03)	0.30 (0.10–0.93)	<0.001
IADL disability	1.00 (Ref)	0.95 (0.22–4.20)	0.85 (0.41–4.89)	0.73 (0.16–3.24)	0.68 (0.30–1.69)	0.042
IADL moderate/severe disability	1.00 (Ref)	0.94 (0.21–4.11)	0.83 (0.39–3.54)	0.73 (0.16–3.24)	0.58 (0.25–2.01)	0.022
EQ-5D score <1	1.00 (Ref)	0.92 (0.52–2.91)	0.83 (0.44–3.52)	0.69 (0.33–2.40)	0.44 (0.34–2.71)	<0.001
**CHONS**						
BADL disability	1.00 (Ref)	0.74 (0.41–1.33)	0.65 (0.35–1.22)	0.58 (0.32–1.08)	0.41 (0.19–0.86)	<0.001
BADL moderate/severe disability	1.00 (Ref)	0.83 (0.38–2.18)	0.99 (0.21–4.66)	1.04 (0.46–2.11)	0.99 (0.35–3.08)	0.882
IADL disability	1.00 (Ref)	0.78 (0.43–1.12)	0.71 (0.40–1.28)	0.62 (0.33–1.14)	0.55 (0.28–1.10)	0.039
IADL moderate/severe disability	1.00 (Ref)	0.95 (0.48–1.87)	0.90 (0.60–1.78)	0.81 (0.45–1.80)	0.72 (0.61–1.32)	0.045
EQ-5D score <1	1.00 (Ref)	0.91 (0.48–1.70)	0.80 (0.46–2.65)	0.61 (0.32–2.45)	0.56 (0.34–2.68)	0.010

*Adjusted for age, gender, nationality, education, marital status, and alcohol drinking*.

*CHCCS, China Hainan Centenarian Cohort Study; CHOCS, China Hainan Oldest-old Cohort Study; ICH, ideal cardiovascular health; OR, odds ratio; BADL, basic ability of daily life; IADL, instrumental ability of daily life*.

Considering there is a close relationship between physical activity and disability, we removed the physical activity from the ICH metrics in the sensitivity analysis. The main results and trends remained unchanged (Appendix Table 4).

## Discussion

This study investigated the distribution of ICH metrics in oldest-old and centenarians and explored the association between the level of ICH metrics and disability and health-related quality of life. The results show that the centenarians and the oldest-old tend to have relatively high level of ICH metrics and there is a strong and independent association between the number of ICH metrics and disability and lower health-related quality of life. It is reported from previous studies that the overall level of ICH metrics among centenarians and the oldest-old was relatively higher than adults or young elderly (20–23). Our results showed that 8.7% of the centenarians and 10.3% of the oldest-old had more than 6 ICH metrics at the ideal level, while the percentage was only 4.3% among those aged ≥45 based on a nationally representative sample of 96,121 Chinese adults (13). The status of smoking and BMI are impacted by alterable behaviors and the percentage of ideal status for these two metrics nearly reach 90%, but the percentage of ideal level of physical activity is still very low. This finding is consistent with the results from some previous research from which it is reported that the population smoking rate increases at the age of 20 through to 60 and then decreases as the population gets older (24, 25). The percentage of ideal level of diet is <40.0%. It is mainly driven by the low consumption of dairy products which is common in China, especially among the aged population (26). The percentage of ideal level of FPG and TC is relatively high whereas the percentage of ideal blood pressure level is low (8.3%). We have seen similar results in previous studies that the glucose and TC levels are well-managed and controlled in an older population (27, 28). This finding may be explained by survival bias. That is, the elderly with metabolic disorders died before the age of 80. In addition, a series of studies show that metabolic abnormalities peak before reaching the age of 80, then blood sugar and lipid levels gradually decline. The mechanism is still unclear, which may be related to nutrition and slow metabolism in the older population (29–31). Hypertension is one of the most important risk factors for cardiovascular diseases. A study of 1.3 million survey results shows that the prevalence of hypertension in China is high and the control rate is low (32). Our study also shows the low percentage of ideal blood pressure levels among all subjects. This suggests it is important to improve proper treatment and control measures of hypertension.

Our results show that as the number of ICH metrics increases, both disability and lower health-related quality of life rate decreases. Some previous research has pointed out that the poor ICH status is associated with an increasing prevalence of cardiovascular diseases, including myocardial infarction, atrial fibrillation, heart failure, stroke, etc. (5, 6). Meanwhile, other researchers suggest that ICH metrics are related with other diseases and symptoms, including diabetes, non-alcoholic fatty liver disease, and depression (8, 9). However, previous research shows little evidence about the association between ICH metrics and disability, especially IADL. We investigated the relationship, and our results indicate that ICH metrics are associated with both BADL and IADL disability, especially moderate to severe disability. The effect of one single metric, either health behavior or health factor, on disability or health-related quality of life is weak, while the effect of the combined metrics is strong. This suggests that the ICH metrics are a good indicator for screening high-risk disability in the elderly (11, 12).

ICH status is not only related to cardiovascular disease prevention, but also to multiple other health outcomes. The correlation between single risk factor and disability is weak, but the correlation between the combination ideal cardiovascular health factors and disability or health-related quality of life shows a statistical difference, and this correlation is more significant for moderate to severe disability.

Our study benefits from some unique aspects. First, this is the first survey about the distribution of ICH metrics and association with both disability and health-related quality of life based on elderly aged over 80 years old. Secondly, most previous studies focused on inpatients or institutionalized elderly, our study recruited community-based participants with a large sample size. In addition, we investigated both BADL and IDAL to understand disability more comprehensively. However, there were several limitations in this study. First, all the participants are from Hainan province, making it hard to generalize across other oldest-old and centenarian groups. Second, the association analysis is based on cross-sectional design that does not provide a strong causal inference. And the application of ICH metrics was modified to accommodate the lifestyle and characteristics of the older population in China. Lastly, the effect of survival bias might be very strong in the study samples, which may have effects on the results.

## Conclusions

The results show that the overall level of ICH metrics of centenarians and oldest-old is relatively good and there is a strong and independent relationship between ICH metrics and both disability and lower health-related quality of life. The results highlight the importance of cardiovascular prevention even at 80 years and above.

## Perspectives

### Core Clinical Competencies in Medical Knowledge

The centenarians and the oldest-old had a better level of ICH metrics. And there was a strong and independent relationship between ICH metrics with both disability and lower health-related quality of life at this age group.

### Translational Outlook

Even at the age of 80 or above, attention should be paid to maintain better ICH status. This was closely related with the self-care and better health-related quality of life, which was very important in the later life.

## Data Availability Statement

The original contributions presented in the study are included in the article/supplementary material, further inquiries can be directed to the corresponding author/s.

## Ethics Statement

The studies involving human participants were reviewed and approved by the Biomedical Ethics Committee of Chinese PLA General Hospital. The patients/participants provided their written informed consent to participate in this study.

## Author Contributions

ML, FK, and SY were involved in statistical analysis and result interpretation. SW and WZ were involved in manuscript drafting. YH was involved in study design, manuscript drafting, and review. All authors contributed to the article and approved the submitted version.

## Conflict of Interest

The authors declare that the research was conducted in the absence of any commercial or financial relationships that could be construed as a potential conflict of interest.

## Publisher's Note

All claims expressed in this article are solely those of the authors and do not necessarily represent those of their affiliated organizations, or those of the publisher, the editors and the reviewers. Any product that may be evaluated in this article, or claim that may be made by its manufacturer, is not guaranteed or endorsed by the publisher.

## Abbreviations

BADL: basic ability of daily life
BMI: body mass index
CI: confidence interval
CHCCS: China Hainan Centenarian Cohort Study
CHOCS: China Hainan Oldest-old Cohort Study
DBP: diastolic blood pressure
FPG: fasting plasma glucose
HDL-C: high density lipoprotein cholesterol
IADL: instrumental ability of daily life
ICH: ideal cardiovascular health
IFG: impaired fasting glucose
LDL-C: low density lipoprotein cholesterol
OR: odds ratio
SBP: systolic blood pressure
TC: total cholesterol
TG: triglyceride
VAS: visual analog scale.
