# Supplementary material for: Ideal Cardiovascular Health in the Oldest-Old and Centenarians and Its Association With Disability and Health-Related Quality of Life
Source: Front Cardiovasc Med. 2021 Aug 20;8:603877. doi: 10.3389/fcvm.2021.603877 (PMC8417589; doi:10.3389/fcvm.2021.603877)
Supplement: Supplementary file 1 [file Table_1.docx]

Appendix table 1. Classifications of BADL and IADL

| Classifications | BADL score | IADL score |
| --- | --- | --- |
| Complete self-care | 100 | 8 |
| Mild disability | 61-99 | 6-7 |
| Moderate disability | 41-60 | 3-5 |
| Severe disability | 0-40 | 0-2 |

BADL, basic ability of daily life; IADL, instrumental ability of daily life;

Appendix table 2. Definition of ICH

| Metric | poor | intermediate | ideal |
| --- | --- | --- | --- |
| Smoking | Current | Former (quit≤12 month) | Never or quit>12 month |
| BMI | BMI≥28 kg/m^2^ | 24 kg/m^2^≤BMI＜28kg/m^2^ | BMI<24kg/m^2^ |
| Physical activity | None or incapable | 1 times/week≤more than 10 minutes’ walk≤4 times/week | more than 10 minutes’ walk≥5 times/week |
| Healthy diet score | 0-1 components | 2-3 components | 4-5 components |
| TC | ≥6.20mmol/L (240mg/dL) | 5.17-6.19 mmol/L(200-239mg/dL) or treated to goal | ＜5.17mmol/L (200mg/dL) without medication of dyslipidemia |
| Blood pressure | SBP≥140mmHg or DBP≥90mmHg | SBP 120-139mmHg or DBP 80-89mmHg, or treated to goal | SBP<120mmHg and DBP<90mmHg without medication or physician diagnosis of hypertension |
| FPG | ≥7.00mmol/L(126 mg/dL) | 5.56-6.99 mmol/L(100-125mg/dL) or treated to goal | <5.56mmol/L (100mg/dL) without medication od physician diagnosis of diabetes |

ICH, ideal cardiovascular health; BMI: body mass index; SBP, systolic blood pressure; DBP, diastolic blood pressure; TC, total cholesterol; FPG, fasting plasma glucose;

Appendix Table 3. Distribution of disability

|  | CHCCS | | | |  | CHONS | | | | p* | Total |
| --- | --- | --- | --- | --- | --- | --- | --- | --- | --- | --- | --- |
|  | male | female | p | subtotal |  | male | female | p | subtotal |  |  |
| BADL |  |  | <0.001 |  |  |  |  | <0.001 |  | <0.001 |  |
| 100(complete self-care) | 29.4 | 13.6 |  | 16.5 |  | 76.6 | 61.3 |  | 67.4 |  | 39.1 |
| 61~ (mild disability) | 46.7 | 56.7 |  | 54.9 |  | 20.9 | 33.9 |  | 28.7 |  | 43.3 |
| 41~ (moderate disability) | 13.9 | 14.2 |  | 14.2 |  | 0.9 | 2.7 |  | 2.0 |  | 8.8 |
| ≤40(severe disability) | 10.0 | 15.5 |  | 14.5 |  | 1.6 | 2.1 |  | 1.9 |  | 8.9 |
| IADL |  |  | <0.001 |  |  |  |  | <0.001 |  | <0.001 |  |
| 8(complete self-care) | 4.4 | 1.3 |  | 1.9 |  | 42.2 | 16.1 |  | 26.6 |  | 12.8 |
| 6~ (mild disability) | 10.0 | 5.2 |  | 6.1 |  | 25.0 | 30.3 |  | 28.2 |  | 15.9 |
| 3~ (moderate disability) | 33.3 | 26.0 |  | 27.3 |  | 20.9 | 32.8 |  | 28.1 |  | 27.7 |
| ≤2(severe disability) | 52.2 | 67.4 |  | 64.7 |  | 11.9 | 20.7 |  | 17.2 |  | 43.6 |

*: p for comparisons between CHCCS and CHONS.

CHCCS, China Hainan Centenarian Cohort Study; CHOCS, China Hainan Oldest-old Cohort Study; BADL, basic ability of daily life; IADL, instrumental ability of daily life;

Appendix Table 4. Multivariable analysis of ICH (excluding physical activity) with disability and health-related quality of lif

| Variables | Number of ICH OR(95%CI) | | | | |
| --- | --- | --- | --- | --- | --- |
|  | ≤2 | 3 | 4 | ≥5 | p |
| CHCCS |  |  |  |  |  |
| BADL disability | 1.00(Ref) | 0.95(0.74-1.23) | 0.83(0.67-1.25) | 0.72(0.54-1.18) | 0.042 |
| BADL moderate/severe disability | 1.00(Ref) | 0.93(0.61-1.42) | 0.66(0.27-1.58) | 0.46(0.29-1.08) | 0.016 |
| IADL disability | 1.00(Ref) | 0.83(0.26-2.64) | 0.88(0.34-1.27) | 0.31(0.06-1.76) | 0.434 |
| IADL moderate/severe disability | 1.00(Ref) | 0.85(0.46-1.58) | 0.69(0.35-1.36) | 0.58(0.18-1.85) | 0.042 |
| EQ-5D score<1 | 1.00(Ref) | 0.82(049-1.37) | 0.78(0.51-1.15) | 0.69(0.37-2.06) | 0.048 |
| CHONS |  |  |  |  |  |
| BADL disability | 1.00(Ref) | 0.96(0.49-1.47) | 0.73(0.47-1.09) | 0.70(0.51-0.96) | 0.036 |
| BADL moderate/severe disability | 1.00(Ref) | 0.57(0.24-1.35) | 0.37(0.13-1.03) | 0.51(0.14-1.92) | 0.268 |
| IADL disability | 1.00(Ref) | 0.95(0.53-1.72) | 0.94(0.60-1.49) | 0.66(0.38-1.25) | 0.035 |
| IADL moderate/severe disability | 1.00(Ref) | 0.96(0.59-1.49) | 0.80(0.64-1.18) | 0.63(0.42-0.95) | 0.039 |
| EQ-5D score<1 | 1.00(Ref) | 0.70(0.48-1.03) | 0.54(0.32-0.93) | 0.50(0.33-0.75) | 0.007 |

Adjusted for age, gender, nationality, education, marital status, alcohol drinking.

CHCCS, China Hainan Centenarian Cohort Study; CHOCS, China Hainan Oldest-old Cohort Study; ICH, ideal cardiovascular health; OR, odds ratio; BADL, basic ability of daily life; IADL, instrumental ability of daily life;
